# Modification of the Protein Amino Acid Content in Hen Eggs as a Consequence of Different Concentrations of Lupine and Soy in Feed

**DOI:** 10.3390/molecules29163727

**Published:** 2024-08-06

**Authors:** Aneta Tomczak, Magdalena Zielińska-Dawidziak, Piotr Klimowicz, Marcin Hejdysz, Sebastian Kaczmarek, Aleksander Siger, Adam Cieślak

**Affiliations:** 1 Department of Food Biochemistry and Analysis, Faculty of Food Science and Nutrition, Poznan University of Life Sciences, ul. Mazowiecka 48, 60-623 Poznan, Poland; aneta.tomczak@up.poznan.pl (A.T.); piotr.klimowicz@up.poznan.pl (P.K.); 2 Department of Animal Nutrition, Faculty of Veterinary Medicine and Animal Science, Poznan University of Life Sciences, Wołyńska 33, 60-637 Poznan, Poland; marcin.hejdysz@up.poznan.pl (M.H.); sebastian.kaczmarek@up.poznan.pl (S.K.); adam.cieslak@up.poznan.pl (A.C.)

**Keywords:** egg, amino acid, soybean meal, blue lupine, hen, experimental fodder

## Abstract

The effect of the diet modification (soybean and lupine addition) on the content of protein and amino acids (AA) in eggs was studied. Both the sampling day and the diet influenced the total protein content. In albumen, the lowest protein content (10.6%) was noted after administering a diet containing 25% lupine; in the same egg the yolk contained the most proteins (16.7%). In the content of nonessential AA (NAA) in egg yolks, differences were noted only for cysteine, with its the highest content in the yolks of the control group. The stable content of essential yolk amino acids (EAA) was observed only for isoleucine, leucine, tryptophan and phenylalanine. The highest contents of EAA and NAA were recorded in the yolks of the control group (~47 and ~53 g/100 g of protein, respectively) and in the group with 25% additions of lupine (~42 and ~51 g/100 g of protein, respectively). AA with constant content in the tested albumens were methionine, tryptophan and alanine. The highest content of EAA (>~42 g/100 g of protein) and NAA (>~62 g/100 g of protein) were determined in albumen of eggs determined in the group with at least 20% additions of lupine. The highest content of EAA for humans delivered eggs from groups 4–6 (with the addition of soy into the diet ≤5%). The protein sources used in the hen diet significantly influenced the content of protein and individual AA in the produced eggs.

## 1. Introduction

Hen egg is an excellent and natural product in human nutrition. It consists of egg white (60%), yolk (30%) and shell (10%); in chemical terms: of water (75%), protein (12%), lipids (12%) [1]. Due to their low price and excellent nutrients composition, eggs have be-come an extremely important animal product around the world [2,3]. It is a basic ingredient of many foodstuffs and therefore plays an important role in human nutrition [4]. The parameters influencing egg quality are: composition, shell quality and egg size. Feeding hens is a primary factor influencing each of these parameters [5]. What is also significant is the hens’ age [6], while breed and breeding system are less important [7,8]. Currently, many studies are focused on the eggs’ composition modifications, depending on the feed components. Mainly, the influence of feed on the alteration of fatty acids in eggs has been studied, but interest is also observed in vitamins, color or elements [5,9,10,11].

Modification of feed mixtures must be strictly controlled to keep nutrient balance. The main ingredients that currently provide energy and protein in the poultry diet are soybean meal and corn [12]. Enormous efforts have been made for years in the EU to replace soybean meal with European sources of protein, both for economic and social reasons [13,14]. Finding a substitute for soy is an important challenge for the animal breeding sector. The protein content in cereals is too low to meet the nutritional requirements of hen feeding [1]. Apart from soybeans, other legumes such us lupine, which has a similar composition and a protein level, are used. The multitude of lupine varieties enables choosing a variety that is free of alkaloids, and simultaneously raw material that is not genetically modified [15,16,17]. The addition of lupine or lupine flour to the diet of laying hens influences both the quality of eggs as well as the health of hens, including their lipid metabolism, reducing blood cholesterol levels and the birds’ mortality [13]. Lupine can only partially replace soybean meal in the diet of laying hens due to its lower level of protein and metabolic energy and the presence of some antinutritional components [13]. Drazbo et al. (2014) [13] indicated that the impact of using lupine in the feeding of laying hens on the quality of eggs has not been sufficiently studied so far. Moreover, the available research focuses on the modification of the lipid profile [6,18] and completely ignores the impact of feed modification on the protein level and amino acid composition of eggs. Some data suggest that oats and barley, unlike corn and wheat, have a positive effect on egg protein [1], as well as that the introduction of fermented feed or protein-rich waste products from the food industry [19,20]. The effect was not observed in the amount of total egg protein, but in the concentration of exogenous amino acids. There is no research on the lupine impact on eggs protein.

In poultry nutrition, post-extraction soybean meal is a very important ingredient. In Poland, searching for local sources of protein, such as lupine, is dictated by the need for more sustainable agriculture. The aim of the presented research was to detect potential differences in the amount of protein and amino acid composition of egg albumen and yolk, depending on the variable addition of lupine (0–25%) and soy (0–15.43%) to fodder. The inclusion of different shares of lupine and soybean in the mixture aimed at verifying which of the levels used would be the most beneficial for the amino acids composition of eggs.

## 2. Results

### 2.1. Performance Results 

During the four-week experiment, there was no statistically significant effect of the dose of narrow-leaved lupine used in the diets on the rate of laying and egg weight (*p* > 0.05). The analysis of feed intake results showed a statistically significant negative effect of the presence of soybean meal and narrow-leaved lupine in the diet on feed intake by laying hens (*p* < 0.05). 

However, varying the dose of narrow-leaved lupine in the diet did not significantly influence the variation in feed intake. In the case of feed conversion ratio (FCR) per kg of eggs, the increasing level of narrow-leaved lupine in the diets for laying hens significantly reduce the value of this parameter (*p* < 0.05) (Table 1).

### 2.2. Total Protein Analysis 

The content of protein in the experimental diet is presented in Table 2. The feed mixtures used had a protein level of approximately 18%. However, the highest content of crude protein was determined in control fodder, not containing soybean or lupine. The feed mixtures did not differ in the content of amino acids; deficient amino acids in feed were supplemented to achieve optimal supply of amino acids to the animals (as it is presented in Section 4.1).

Both the day of egg collection (*p* < 0.0001) and the diet used in hen feeding (*p* < 0.0001) influenced the total protein content in yolk, while in egg albumen, protein content depended on used diet only (*p* < 0.0001). The average total protein content in egg yolk was 16.3 ± 0.5% [g/100 g of yolk], and the lowest content (16.0%) was noted for the yolk obtained after feeding hens with the diet containing the highest soybean meal addition and no lupine (fodder No. 1) (Table 3A). In the case of egg whites, a significant decrease in protein content was observed for eggs obtained from the hens fed with feed containing the highest lupine addition (25%) and no soy (fodder No. 5). In this group the average total protein content was 10.6 ± 0.0%, while in the remaining experimental groups it was on average 11.5 ± 0.3% (Table 3B).

### 2.3. Amino Acids Content 

Twenty protein amino acids were analyzed in both egg white and egg yolk, on the first and last days of eggs sampling. The tables below (No. 4–7) present the average results of the content of exogenous and nonessential amino acids in yolks and albumen. No differences in the content of nonessential amino acids in yolk were noted after the application of the six studied feeds, except for cysteine, the content of which was the highest in yolks of control group (43–64% more than for experimental group).

The lowest content of total nonessential amino acids was recorded for yolk samples when hens were fed with the first mixture (containing 25% of soy and no lupine), while its highest content of total nonessential amino acids was recorded for eggs obtained from control (C) and groups with 25% addition of lupine (group 5) (see Table 4 below).

In the case of the essential amino acids of yolks, only in four of them (Ile, Leu, Phe, Trp) was there no significant differences in the content depending on the feed used (Table 5). The content of Lys varied in the range 5.24–8.08 g/100 g of protein, Thr 4.64–6.62 g/100 g of protein, Val 4.46–6.19 g/100 g of protein, His 2.58–3.87 g/100 g of protein and Met 1.25–2.48 g/100 g of protein. The highest content of total essential amino acids was noted in eggs from group 5 (25% of lupine) and C (41.86 and 47.22 g/100 g of protein, respectively).

**Figure 1:**
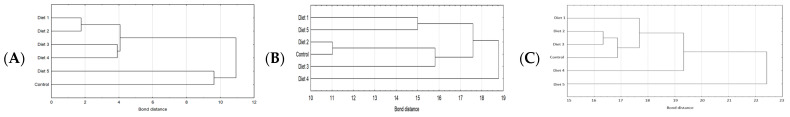
Cluster analysis of the amino acids composition in egg yolk (**A**), white (**B**), and whole egg (**C**) depending on the diet used.

Cluster analysis showed that due to the content of amino acids in the yolks, samples 1 and 2 (from feeding hens with fodders containing 15.43% of soy, and 11% of soy plus 10% of lupine, respectively) differed the most from the control and the content of amino acids in these groups was the smallest. Sample 5 (from the group fed without soy addition) remained similar to the control, and these two groups (5 and C) were characterized by the highest content of amino acids (Figure 1).

In the egg albumens studied, the differences were noted both in the content of essential and nonessential amino acids (Table 6 and Table 7). The only amino acids the content of which was constant in the samples tested were two essential amino acids: methionine (average content about 2.38 g/100 g of protein) and tryptophan (~1.32 g/100 g of protein), and nonessential amino acid, i.e., Ala (~14.60 g/100 g of protein). The content of nonessential amino acids varied in the ranges: Ser 5.21–7.89 g/100 g of protein, Arg 4.37–6.67 g/100 g of protein, Asp + Asn 2.65–4.51 g/100 g of protein, Glu + Gln 8.80–15.10 g/100 g of protein, Pro 4.48–6.83 g/100 g of protein, Tyr 2.92–4.10 g/100 g of protein and Cys 2.11–4.14 g/100 g of protein. However, essential amino acids varied as follows: Ile 3.45–5.82 g/100 g of protein, Leu 6.20–9.59 g/100 g of protein, Lys 3.84–6.00 g/100 g of protein, Thr 3.75–5.38 g/100 g of protein, Val 4.18–8.02 g/100 g of protein, His 1.87–3.70 g/100 g of protein and Phe 4.96–7.22 g/100 g of protein. The highest content of total nonessential and essential amino acids was noted in the albumen of eggs obtained after feeding with diets 4 and 5 (containing at least 20% lupine).

Cluster analysis confirmed that sample 2 (eggs after hen feeding with 11.0% of soy and 10% of lupine) differed the least from the control, while sample 4 (obtained after hen feeding with 5% of soy and 20% of lupine) was the most different in the content of amino acids in the albumen (Figure 1).

Cluster analysis of the egg albumen and yolk together suggested that the eggs obtained after feeding with diets 2 and 3 were the most comparable in amino acid composition and simultaneously they were the most similar to the control (C) eggs, while eggs from group 5 differed the most from the controls.

**Figure 2:**
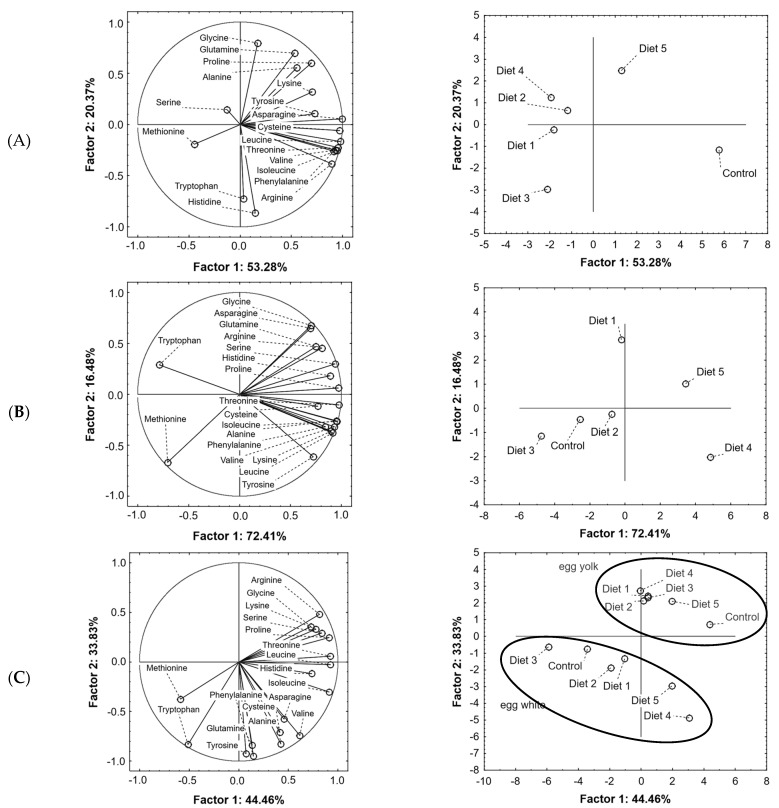
Principal components analysis (PCA) of amino acids content in egg yolk (**A**), white (**B**), yolk and white (**C**).

The analysis of yolk amino acid composition with PCA showed that the first two factors explained 73.65% of the total variance (53.28% and 20.37%, respectively) (Figure 2A). The F1 factor was positively correlated with Arg (r = 0.89), Asp (r = 0.99), Thr (r = 0.96), Val (r = 0.93), Ile (r = 0.95), Leu (r = 0.98), Phe (r = 0.91) and Cys (r = 0.97). However, the F2 factor was negatively correlated with His (r = −0.87) and Trp (r = −0.73) and positively with Gly (r = 0.79) and Glu + Gln (r = 0.70). The diet used (additions of soybean and lupine) significantly influenced the amino acid composition of the yolks (Figure 2A). A decrease in the content of Arg, Asp, Thr, Val, Ile, Leu, Phe and Cys was observed compared to the control feed. However, diet 5 (with the highest addition of lupine) was characterized by an increase in the content of Glu, Ala, Pro, Lys in egg yolks.

In the case of egg whites, the first two factors explain the variability of the obtained data in approximately 88.89% (72.41% and 16.48%, respectively) (Figure 2B). The F1 was positively correlated with the content of all amino acids (r = 0.70–0.97), except negatively correlated with Met (r = −0.71) and Trp (r = −0.79) content (Figure 2A). The F2 was positively correlated with the content of Gly (r = 0.67) and Asn (r = 0.65), while negatively correlated with Tyr (r = −0.61) and Met (r = −0.67) (Figure 2B). PCA results confirmed that the amino acid composition of egg whites differed significantly depending on the feed used (Figure 2B) and compared to the control sample, the greatest differences were noted for egg whites obtained from hens fed with diets 1, 4 and 5 (i.e., with the two highest soybean addition and with two the highest lupine additions). However, diets 2 (with 11% of soy and 10% of lupine) and 3 (8.6% of soy and 15% of lupine) did not cause such significant changes in the amino acid composition of chicken egg whites.

Figure 2C shows the PCA analysis for the white and yolks amino acids tested together. The analysis showed that the first two factors explain 78.29% of the total variance (F1—44.46% and F2—33.83%). The F1 factor was positively correlated with Ser (r = 0.84), Arg (r = 0.81), Thr (r = 0.92), Pro (r = 0.92), Ile (r = 0.91) and Leu (r = 0.92). It was negatively correlated with Met (r = −0.59) and Trp (r = −0.51). However, the F2 factor was negatively correlated with Glu + Gln (r = −0.84) and Ala (r = −0.82), Tyr (r = −0.92), Phe (r = −0.95) and Trp (r = −0.83) (Figure 2C). The most differentiating amino acids between egg white and yolk were Ser, Arg, Gly, Lys (higher content in egg yolks), Met and Trp (higher content in egg whites).

## 3. Discussion

Research on the impact of fodder egg quality is of wide interest due to the possibility of designing eggs with specific nutritional and health-promoting features. Egg producers are concerned with possible modification of feed ingredients which are economically beneficial and consistent with the principles of sustainable development. Feed ingredients in poultry diets are carefully selected in terms of nutritional value which they provide to the animals. A properly balanced diet is intended to ensure the bioavailability of the nutrients needed by the hen [21,22].

The composition of individual fodder mixtures used in the presented experiment was carefully selected both in terms of energy and nutrients supply. The total protein content was at a similar level (Table 2), and the amino acids were supplemented to ensure an adequate supply of amino acids with the diet (as it is presented in Section 4.1). According to the literature data, the protein level in feed for laying hens should range between 15 to 20% [12], which was ensured by the prepared mixtures. The birds performed very well; however, C treatment birds were characterized by the lowest feed intake (*p* < 0.05), which could be explained by the highest availability of nutrients. The raw materials used (potato protein, corn) are highly available for layers.

The application of lupine seeds in hen feeding has been studied before, but it usually concerned the impact on the egg yolk lipids and vitamin composition [23,24,25]. The influence of this fodder component on proteins of egg albumen and yolks was omitted [13]. Studies on modification of this egg constituent are very important, because chicken egg protein is considered standard in terms of amino acid composition in older children, adolescent and adult human nutrition [26].

According to 2018 data from the ‘USD A National Nutrient Database’, one large egg contains 6.3 g of protein, divided between yolk and albumen (3.6 g in egg white and 2.7 g in egg yolk) [27]. Per 100 g, egg white contains on average 11% protein and yolk—about 16%. In terms of the data concerning the composition, no studied egg differed from those described in the literature. However, in egg white obtained from the group 5 (i.e., fed with the fodder containing 25% of lupine and no soy), the content of crude protein was significantly lower than in other groups (Table 3), and this tendency was stable during the seven days of eggs sampling. At the same time, the protein content was the highest in yolks from the same group. Slight differences in the protein content in eggs were also shown by Revathy (2020) [22], where hens were fed with cooked rice. Previously, it has been shown that lupine oligosaccharides, NSPs and NDF could affect the availability of nutrients for birds [24,25]. It could be hypothesized that observed differences in egg protein content were an effect of different availability and deposition of nutrients. However, in this trial, digest viscosity or oligosaccharides content were not determined.

A two-factor analysis of variance showed that both the diet used and the day of the egg collection had a statistically significant effect on the amino acid content of the sample studied (white and yolk) (*p* < 0.05). It was also found that there was a statistically significant interaction between the two factors (*p* < 0.05) (Table S1). From the producer and consumer point of view, the day of egg collection has no impact on purchasing decisions; thus, these results have not been discussed. It is important to gain knowledge of how the diet composition affects the amino acid content in the eggs obtained, regardless of the day of collection (after the adaptation period to a given feed).

To sum up the analysis of the amino acids content, it can be concluded that manipulation on the protein source in fodder through variable addition of soybean meal and blue lupine significantly affects the level of amino acids. However, it mainly influences the modification of the amount of amino acids in the egg white rather than in the yolk. Mori et al., 2020 [19] also observed variable amino acids composition in eggs depending on the composition of the feed and the breed of chickens, while Kawamura et al., (2023) [28] saw it as a consequence of the combined action of the feed and housing system.

From a nutritional point of view, the content of essential amino acids in eggs (as a foodstuff) is of the most importance. Thus, the composition of these amino acids in studied egg parts was compared to the reference protein [26] (Table S2).

Only lysine content in the albumen of eggs obtained from the first three experimental groups (i.e., after feeding with diet containing 15.43% of soy and no lupine, 11% of soy and 10% of lupine and 8,6% of soy and 15% of lupine) was significantly below the recommended level (marked in gray in Table 7); control albumen was also slightly behind. However, taking into account the fact that egg yolk provides more protein, the lysine level will also be in line with the recommendations for those eggs consumed in whole (Table S2). Nutritionally, whole eggs from hens fed with each mixture provide all the most important amino acids in the right proportions and are a source of complete protein.

It is difficult to replace post-extraction soybean meal in poultry nutrition. Due to the amino acid composition and energy content in lupine, it is most advantageous to use mixtures of lupine and soy. The eggs with the biggest nutritional value for humans were those obtained from hens fed diets 4, 5 and C (containing less than 5% of soybean meal) as these eggs delivered the highest amount of essential amino acids (Table S3). However, in the case of animal nutrition, the administration of such diet is not justified, due to, among others, economic reasons, including the availability and price of raw materials.

Based on the presented research, it can be expected that even if the feed used did not affect the hens’ egg production and egg weight, due to the differences found in the content of individual amino acids, variability in the content of individual protein fractions and morphotic albumen parts may probably be observed. It is consistent with previously presented studies on the bioactive proteins content, when manipulating the feed composition showed significant impact on the activity of cystatin, lysozyme, antitrypsin and anti-chymotrypsin of eggs [28]. Research on the protein profile of the obtained eggs will be continued.

## 4. Materials and Methods

**Figure 3:**
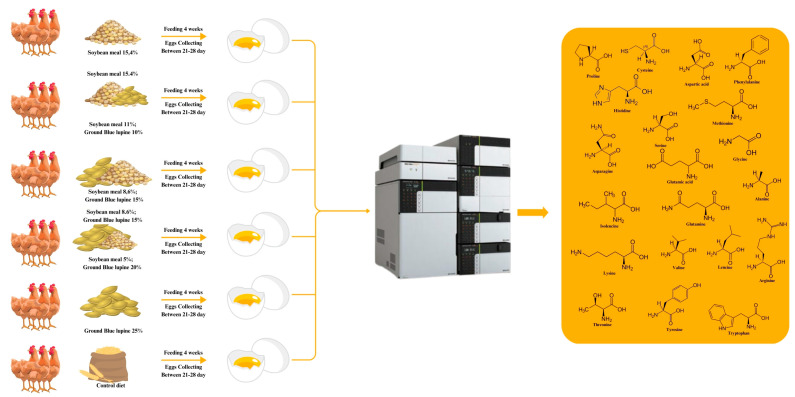
The experiment scheme.

The experiment scheme is presented in Figure 3.

### 4.1. Laying Hens Experiment 

The four-week-long experiment included 360 Hy-Line brown hens at the peak of laying (the age of 22 weeks), with a body weight of approximately 1.7 kg ± 0.5. The hens were delivered by a registered breeding operation with a pedigree. Three randomly chosen animals were kept in one cage and fed one of six diets with variable soybean meal and ground blue lupine seeds with low alkaloid content (Sonet variety). The composition of the feed is presented in Table 8. There were 20 repetitions for each group. The feed was prepared in accordance with the Poultry Nutrition Standards [23,29,30]. Birds had free access to water, and were fed with nitrogenous and isoenergetic fodder in a mash form. The 14 h light/10 h dark cycle was provided. After 21 days of hens’ adaptation to the experimental diet, eggs were collected for the following seven days at the same time in the evening. The experiment was fully supervised by trained, educated and experienced staff. In the study, the following parameters were examined for each week: laying rate, egg weight, feed intake and feed conversion ratio. This type of research did not require approval by the Local Ethical Commission. The research was carried out in accordance with the U.K. Animals (Scientific Procedures) Act, 1986 and associated guidelines, EU Directive 2010/63/EU for animal experiments, and the National Research Council’s Guide for the Care and Use of Laboratory Animals.

### 4.2. Legume Seed Analysis

As a variable feed component influencing the protein expression, commercially available soybean meal (purchased for feed purposes) and blue lupine seeds (*Lupinus angustifolius*, Sonet variety) were used. The seeds used were analyzed in duplicate for dry matter (DM) and crude protein (CP), using AOAC methods 934.01, 976.05, respectively [31]. The neutral detergent fiber (NDF) was determined with heat-stable amylase and expressed inclusive of residual ash using the 973.18 AOAC method [32]. Starch content in seeds was determined using a diagnostic assay kit for agricultural industries [33] on the basis of use of thermostable α-amylase and amyloglucosidase. Alkaloids were extracted from the lupin meal via trichloroacetic acid and methylene chloride and the amounts were determined via gas chromatography (Shimadzu GC17A, San Jose, CA, USA) using a capillary column (Phenomenex, Torrance, CA, USA). The oligosaccharides in meals were extracted and analyzed via high-resolution gas chromatography, as described by Zalewski et al., 2001 [34]. The NSP (non-starch polysaccharides) content in meals was determined via gas-liquid chromatography (neutral sugars) and colorimetry (uronic acids). The procedure for neutral sugars was as described by Englyst and Cummings and applied with some modifications [35]. Uronic acids were marked using the procedure described by Scott, 1979 [36]. Soluble NSP were calculated as the difference between NSP and NDF [37]. The results expressed in g/kg of dry matter (DM) are summarized in Table 9.

### 4.3. Sample Preparation

After breaking the eggs, ten random egg whites and yolks from each day and each experimental group were pooled into one sample and frozen. Samples were stored in tightly closed plastic containers at −18 °C before the analysis.

### 4.4. Protein Quantification

The total protein content was studied in the fodders with albumen and yolk samples collected during seven last days of the experiment, via the Kjeldahl method. The analysis was performed in accordance with AOAC 925.31 [38]. The protein conversion factor used was 6.25.

### 4.5. UHPLC Method

The analysis of the amino acid composition was performed with the UHPLC method for samples from the first and seventh day of eggs collection. Initially, acidic hydrolysis (110 °C, 23 h) and oxidative hydrolysis (4 °C, 16 h and 100 °C, 2 h) were performed via AOAC Official Method 994.12 Amino Acids in Feeds [39]. Acid hydrolysis allows the determination of most protein amino acids (L-Alanine (Ala), L-Arginine (Arg), L-Aspartic Acid (Asp) + L-Asparagine (Asn), L-Glutamic Acid (Glu) + L-Glutamine (Gln), L-Leucine (Leu), L-Lysine (Lys), L-Serine (Ser), L-Threonine (Thr), L-Tyrosine (Tyr), L-Valine (Val), L-Histidine (His), L-Isoleucine (Ile), L-Phenylalanine (Phe), L-Proline (Pro), Glycine (Gly). In turn, oxidative hydrolysis was used to determine sulfur amino acids: L-Methionine (Met) and L-Cystine (Cys) (Tomczak et al., 2018) [40]. After hydrolysis, the samples were subjected to amino acid derivatization (using AccQ • Tag reagents, No. 186003836, Waters, Milford, MA, USA) according to the protocol. The prepared samples were analyzed using a UHPLC chromatograph (UHPLC, Shimadzu Nexera 2.0, Kyoto, Japan) equipped with a binary solvent manager, autosampler, column heater and PDA detector (Kyoto Electronics Manufacturing Co., Ltd., Kyoto, Japan). A column dedicated to amino acids was used (AccQ-Tag Ultra C18 1.7 μm (2.1 mm) ID. × 100 mm, particles 1.7 µm, water). The column temperature was 55 °C, the flow was 0.6 mL/min, a nonlinear separation gradient was used and created by mixing Eluent A and Eluent B AccQ • Tag Ultra (Waters). One microliter of sample was used for analysis at 260 nm [40]. To determine total tryptophan, the sample was hydrolyzed in an alkaline medium using a saturated barium hydroxide solution and heated to 110 °C for 20 h. The analysis was performed using an internal standard. Tryptophan in the extract was determined via HPLC with fluorescence detection. Tryptophan was determined separately via high-performance liquid chromatography using fluorescence detection (HPLC-FLD) in accordance with Commission Regulation (EU) No. 152/2009 of 27 January 2009, Annex III G. The results are expressed in g per 16 g N, i.e., in g per 100 g of protein.

### 4.6. Statistical Analysis

Results are presented as an average ± standard deviation from three replicates of each experiment. An analysis of variance (ANOVA) was performed, followed by a Tukey post hoc test at a significance level of *p* < 0.05 to compare means. Multivariate statistical analysis of the mean values obtained for egg white and yolk and eighteen variables (n = 18)—the content of individual amino acids—was carried out using principal component analysis (PCA) and hierarchical cluster analysis (HCA). The statistical analysis was performed using Statistica (data analysis software system), version 13 (TIBCO Software Inc. 2017, Palo Alto, CA, USA).

## 5. Conclusions

Currently, modifying the composition of eggs via the application of different diets for hens is very popular, although there are few scientific works on the aspect of egg proteins and their modification. The results of the presented experiment indicate the significant impact of the blue lupine and soybean meal addition to the diet of laying hens both on the protein content and amino acids composition of eggs. The variable content of these legumes in the diet did not have a significant effect on egg production and egg weight. The presence of lupine in feed had a negative impact on feed intake. Both the day of egg collection and the diet used in feeding the hens influenced the total protein content in egg yolk and egg white. The addition of blue lupine to feed at the level of 25%, with the simultaneous absence of soy, resulted in the lowest protein content in hen egg white, while the highest in egg yolk.

Regardless of the feed used, the level of exogenous amino acids in the yolk was consistent with the reference protein. In the case of egg albumen, some lysine deficiencies were found in eggs obtained from hens fed with higher doses of soybean meal. These differences were not significant for a whole egg, due to the mass proportions between the egg white and yolk. Obtained results suggest variability in the morphotic albumen parts in the studied eggs.

## Figures and Tables

**Table 1:** Performance results of laying hens—average results from 25 to 28 weeks of experiment.

Experimental Group	Egg Weight (g)	Laying Rate (%)	Feed Intake (g/day)	FCR ^1^ (kg·kg^−1^ Eggs)
1	60 ± 2 ^a,^*	95 ± 7 ^a^	119 ± 8 ^b^	2.1 ± 0.3 ^c^
2	61 ± 2 ^a^	97 ± 2 ^a^	116 ± 5 ^b^	2.1 ± 0.1 ^c^
3	61 ± 2 ^a^	96 ± 3 ^a^	119 ± 5 ^b^	2.0 ± 0.1 ^b,c^
4	60 ± 2 ^a^	97 ± 4 ^a^	115 ± 4 ^b^	2.0 ± 0.1 ^a,b^
5	60 ± 2 ^a^	96 ± 4 ^a^	119 ± 6 ^b^	2.0 ± 0.2 ^b,c^
C	60 ± 2 ^a^	97 ± 3 ^a^	110 ± 6 ^a^	1.9 ± 0.1 ^a^

^1^ FCR—feed conversion ratio. * values marked with different letters in the columns show statistically significant differences at *p* < 0.05.

**Table 2:** Total protein content in experimental fodder [%].

Experimental Diets	Protein Content
1	20 ± 1 ^c,^*
2	17.7 ± 0.3 ^a^
3	18.4 ± 0.9 ^a,b^
4	18.1 ± 0.1 ^a^
5	18.4 ± 0.6 ^a,b^
C	20 ± 1 ^c^

* values marked with different letters (^abc^) are significantly different (*p* < 0.05).

**Table 3:** Total protein content in hen egg yolk (A) and albumen (B). Results from seven consecutive days of eggs’ collecting (21–28 day of feeding hens with experimental fodders).

**(A)**								
**Diet**	**Protein Content in Egg Yolk [%]**							
	1 ^C,^*	**2 ^C^**	**3 ^A^**	**4 ^A,B^**	**5 ^B,C^**	**6 ^B,C^**	**7 ^B,C^**	**Mean Value**
1	16.1 ± 0.2	17.2 ± 0.3	15.36 ± 0.07	16.3 ± 0.1	15.73 ± 0.03	15.67 ± 0.06	15.4 ± 0.2	16.0 ± 0.6 ^a,^*
2	16.9 ± 0.1	16.5 ± 0.6	15.35 ± 0.05	15.39 ± 0.06	16.8 ± 0.3	16.57 ± 0.03	16.9 ± 0.4	16.4 ± 0.7 ^a,b^
3	16.8 ± 0.3	16.67 ± 0.04	16.6 ± 0.3	15.7 ± 0.1	16.5 ± 0.2	16.4 ± 0.2	16.4 ± 0.6	16.4 ± 0.4 ^a,b^
4	16.5 ± 0.3	16.3 ± 0.2	15.75 ± 0.07	16.79 ± 0.07	16.6 ± 0.2	16.00 ± 0.09	16.7 ± 0.2	16.4 ± 0.4 ^a,b^
5	16.76 ± 0.02	16.47 ± 0.02	16.5 ± 0.2	16.2 ± 0.1	16.61 ± 0.06	17.44 ± 0.01	16.98 ± 0.01	16.7 ± 0.4 ^c^
C	16.6 ± 0.1	16.22 ± 0.06	15.69 ± 0.03	15.5 ± 0.1	16.7 ± 0.1	16.5 ± 0.2	16.5 ± 0.1	16.2 ± 0.5 ^a^
**(B)**								
**Diet**	**Protein Content in Egg White [%]**							
	1 ^A,^*	**2 ^A^**	**3 ^A^**	**4 ^A^**	**5 ^A^**	**6 ^A^**	**7 ^A^**	**Mean Value**
1	11.3 ± 0.2	11.5 ± 0.3	11.7 ± 0.2	11.4 ± 0.2	11.40 ± 0.04	11.90 ± 0.01	11.26 ± 0.08	11.5 ±0.3 ^b^
2	11.85 ± 0.05	11.52 ± 0.08	11.43 ± 0.06	11.26 ± 0.06	10.94 ± 0.03	11.1 ± 0.1	11.8 ± 0.1	11.4 ± 0.3 ^b^
3	11.56 ± 0.08	11.98 ± 0.01	11.3 ± 0.1	11.6 ± 0.2	11.1 ± 0.1	11.18 ± 0.04	11.5 ± 0.2	11.5 ± 0.3 ^b^
4	11.4 ± 0.3	11.5 ± 0.6	11.8 ± 0.1	11.81 ± 0.03	11.32 ± 0.06	11.5 ± 0.1	11.9 ± 0.7	11.6 ± 0.4 ^b,c^
5	10.76 ± 0.04	10.47 ± 0.00	10.41 ± 0.00	10.48 ± 0.07	10.61 ± 0.07	10.62 ± 0.06	10.85 ± 0.06	10.6 ± 0.2 ^a^
C	11.8 ± 0.2	11.6 ± 0.2	11.72 ± 0.04	11.67 ± 0.04	11.67 ± 0.08	11.82 ± 0.06	12.2 ± 0.2	11.8 ± 0.2 ^c^

* Capital letters inform about significant differences (*p* < 0.05) among the samples resulting from day of eggs collection, lower case letters inform about differences resulting from the diet used.

**Table 4:** Content of nonessential amino acids in egg yolk [g/100 g of protein]. Y—yolk; 1–5, C—symbol of a diet.

Sample	Amino Acid								Total NAA ***
	SER	ARG **	ASP + ASN	GLU + GLN	ALA	PRO	TYR	CYS	
Y1	7.9 ± 0.3 ^a,^*	7.4 ± 0.3 ^a^	9.6 ± 0.2 ^a^	11.4 ± 0.4 ^a^	4.7 ± 0.2 ^a^	3.6 ± 0.1 ^a^	1.5 ± 0.1 ^a^	1.19 ± 0.2 ^a^	37.69
Y2	8.0 ± 0.3 ^a^	7.3 ± 0.2 ^a^	9.9 ± 0.3 ^a^	11.4 ± 0.2 ^a^	4.8 ± 0.1 ^a^	4.1 ± 0.1 ^a^	1.7 ± 0.1 ^a^	1.2 ± 0.1 ^a,b^	48.4
Y3	8.3 ± 0.3 ^a^	7.6 ± 0.4 ^a^	9.4 ± 0.3 ^a^	11.0 ± 0.6 ^a^	4.7 ± 0.1 ^a^	3.9 ± 0.0 ^a^	1.4 ± 0.3 ^a^	0.8 ± 0.1 ^a^	38.01
Y4	7.6 ± 0.1 ^a^	7.0 ± 0.5 ^a^	9.7 ± 0.5 ^a^	11.2 ± 0.2 ^a^	4.7 ± 0.1 ^a^	4.0 ± 0.2 ^a^	1.5 ± 0.4 ^a^	0.9 ± 0.2 ^a^	46.6
Y5	8.6 ± 0.3 ^a^	7.6 ± 0.1 ^a^	10.6 ± 0.1 ^a^	12.0 ± 0.5 ^a^	5.1 ± 0.2 ^a^	4.2 ± 0.1 ^a^	1.5 ± 0.2 ^a^	1.3 ± 0.2 ^a,b^	50.9
YC	7.7 ± 0.2 ^a^	8.8 ± 0.3 ^a^	12.2 ± 0.2 ^a^	11.6 ± 0.1 ^a^	4.9 ± 0.1 ^a^	4.1 ± 0.1 ^a^	1.8 ± 0.2 ^a^	2.3 ± 0.1 ^b^	53.4

* Letters in a column show statistically significant differences at *p* < 0.05. ** Conditionally essential amino acid. *** NAA-nonessential amino acids.

**Table 5:** Content of essential amino acids in egg yolk [g/100 g of protein]. Y—yolk, 1–5, C—symbol of diet.

Sample	Amino Acid									Total EAA ***
	ILE	LEU	LYS	THR	VAL	HIS **	PHE	TRP	MET	
Y1	4.5 ± 0.1 ^a,^*	8.00 ± 0.00 ^a^	5.4 ± 0.2 ^a^	5.0 ± 0.3 ^a^	4.5 ± 0.3 ^a^	3.5 ± 0.1 ^b,c^	4.1 ± 0.1 ^a^	1.1 ± 0.1 ^a^	2.0 ± 0.1 ^a,b^	38.10
Y2	4.3 ± 0.3 ^a^	8.0 ± 0.2 ^a^	5.5 ± 0.1 ^a^	4.9 ± 0.2 ^a^	4.5 ± 0.5 ^a^	3.2 ± 0.3 ^a,b,c^	4.1 ± 0.2 ^a^	1.1 ± 0.2 ^a^	2.5 ± 0.2 ^b^	38.10
Y3	4.5 ± 0.4 ^a^	7.9 ± 0.3 ^a^	5.8 ± 0.5 ^a^	4.8 ± 0.4 ^a^	5.0 ± 0.5 ^a,b^	3.9 ± 0.2 ^c^	4.2 ± 0.2 ^a^	1.2 ± 0.2 ^a^	2.4 ± 0.4 ^b^	39.70
Y4	4.2 ± 0.1 ^a^	7.7 ± 0.2 ^a^	5.2 ± 0.1 ^a^	4.6 ± 0.1 ^a^	4.7 ± 0.3 ^a,b^	2.6 ± 0.5 ^a^	4.3 ± 0.4 ^a^	1.1 ± 0.1 ^a^	1.3 ± 0.2 ^a^	35.70
Y5	4.8 ± 0.4 ^a^	8.4 ± 0.3 ^a^	8.1 ± 0.4 ^b^	5.1 ± 0.2 ^a^	5.2 ± 0.5 ^a,b^	2.96 ± 0.00 ^a,b^	4.20 ± 0.00 ^a^	1.1 ± 0.1 ^a^	2.0 ± 0.1 ^a,b^	41.86
YC	5.7 ± 0.2 ^a^	10.1 ± 0.1 ^a^	7.1 ± 0.2 ^b^	6.6 ± 0.1 ^b^	6.2 ± 0.3 ^b^	3.56 ± 0.00 ^b,c^	5.36 ± 0.00 ^a^	1.2 ± 0.2 ^a^	1.4 ± 0.5 ^a^	47.22

* Letters in a column show statistically significant differences at *p* < 0.05. ** Conditionally essential amino acid. *** EAA-nonessential amino acids.

**Table 6:** Content of nonessential amino acids in egg white [g/100 g of protein]. W—white. 1–5, C—symbol of diet.

Sample	Amino Acid								Total NAA ***
	SER	ARG **	ASP + ASN	GLU + GLN	ALA	PRO	TYR	CYS	
W1	7.4 ± 0.3 ^c,^*	6.7 ± 0.5 ^b^	4.1 ± 0.4 ^b,c^	15.1 ± 0.6 ^c^	16.3 ± 0.2 ^a^	5.1 ± 0.1 ^a,b^	3.5 ± 0.2 ^a,b^	2.1 ± 0.1 ^a^	60.3
W2	6.9 ± 0.3 ^a,c^	6.4 ± 0.3 ^b^	3.2 ± 0.2 ^a,b^	9.7 ± 0.1 ^a^	13.6 ± 0.5 ^a^	5.2 ± 0.2 ^a,b^	3.2 ± 0.2 ^a,b^	2.6 ± 0.4 ^a^	50.8
W3	5.2 ± 0.2 ^a^	4.4 ± 0.2 ^a^	2.7 ± 0.1 ^a^	8.8 ± 0.1 ^a^	12.7 ± 0.2 ^a^	4.5 ± 0.1 ^a^	2.9 ± 0.1 ^a^	2.2 ± 0.1 ^a^	43.4
W4	7.89 ± 0.00 ^c^	6.41 ± 0.00 ^b^	3.5 ± 0.1 ^a,b,c^	12.8 ± 0.1 ^a,b,c^	16.0 ± 0.2 ^a^	6.8 ± 0.1 ^c^	4.0 ± 0.1 ^b^	4.1 ± 0.1 ^b^	61.5
W5	7.8 ± 0.2 ^c^	6.5 ± 0.3 ^b^	4.5 ± 0.3 ^c^	14.3 ± 1.0 ^b,c^	17.4 ± 0.8 ^a^	6.0 ± 0.2 ^b,c^	4.1 ± 0.2 ^b^	2.5 ± 0.3 ^a^	63.1
WC	5.8 ± 0.5 ^a,b^	5.7 ± 0.6 ^a,b^	3.0 ± 0.3 ^a,b^	10.1 ± 0.1 ^a,b^	11.7 ± 0.6 ^a^	5.20 ± 0.00 ^a,b^	3.3 ± 0.2 ^a,b^	2.4 ± 0.2 ^a^	47.2

* Letters in a column show statistically significant differences at *p* < 0.05. ** Conditional essential amino acid. *** NAA-nonessential amino acids.

**Table 7:** Content of essential amino acids in egg white [g/100 g of protein]. W—white. 1–5, C—symbol of diet.

Sample	Amino Acid									Total EAA
	ILE	LEU	LYS	THR	VAL	HIS **	PHE	TRP	MET	
W1	4.1 ± 0.1 ^a,^*	6.8 ± 0.3 ^a,b^	4.2 ± 0.1 ^a,b^	4.2 ± 0.2 ^a,b^	5.1 ± 0.1 ^a,b^	3.3 ± 0.2 ^b,c^	5.4 ± 0.3 ^a,b^	1.4 ± 0.1 ^a^	2.1 ± 0.2 ^a^	36.6
W2	4.24 ± 0.00 ^a,b^	7.0 ± 0.1 ^a,b^	4.2 ± 0.2 ^a,b^	4.2 ± 0.2 ^a,b^	5.7 ± 0.1 ^b,c^	3.3 ± 0.1 ^b,c^	6.6 ± 0.5 ^b,c^	1.4 ± 0.1 ^a^	2.5 ± 0.1 ^a^	39.14
W3	3.5 ± 0.1 ^a^	6.2 ± 0.2 ^a^	3.8 ± 0.1 ^a^	3.4 ± 0.1 ^a^	4.2 ± 0.2 ^a^	1.9 ± 0.1 ^a^	5.0 ± 0.3 ^a^	1.36 ± 0.00 ^a^	2.8 ± 0.2 ^a^	32.16
W4	5.8 ± 0.1 ^c^	9.59 ± 0.00 ^c^	6.0 ± 0.1 ^c^	5.4 ± 0.1 ^c^	8.02 ± 0.00 ^d^	3.70 ± 0.00 ^c^	7.2 ± 0.1 ^c^	1.2 ± 0.1 ^a^	2.3 ± 0.1 ^a^	49.21
W5	5.0 ± 0.4 ^b,c^	8.0 ± 0.4 ^b^	5.1 ± 0.5 ^b,c^	5.2 ± 0.1 ^b,c^	6.3 ± 0.4 ^c^	3.4 ± 0.2 ^b,c^	6.2 ± 0.5 ^a,b,c^	1.2 ± 0.1 ^a^	2.2 ± 0.1 ^a^	42.6
WC	4.1 ± 0.1 ^a^	6.9 ± 0.2 ^a,b^	4.4 ± 0.1 ^a,b^	3.8 ± 0.3 ^a^	5.0 ± 0.3 ^a,b^	2.7 ± 0.1 ^b^	5.1 ± 0.4 ^a^	1.4 ± 0.1 ^a^	2.5 ± 0.1 ^a^	35.9

* Letters in a column show statistically significant differences at *p* < 0.05. ** Conditional essential amino acid. *** EAA-nonessential amino acids. After feeding with diet containing 15.43% of soy and no lupine, 11% of soy and 10% of lupine and 8,6% of soy and 15% of lupine) was significantly below the recommended level (marked in grey).

**Table 8:** Composition of feed prepared for a feeding experiment conducted for 6 groups of laying Hy-line hens, fed for 4 weeks of the experiment.

Component	1	2	3	4	5	C
Wheat (CP 118)	58.15	53.67	50.28	48.37	47.98	30.00
Corn (CP 94)	-	-	-	-	-	23.19
Soybean meal	15.43	11.00	8.60	5.00	-	-
Blue lupine	-	10.00	15.00	20.00	25.00	-
Peas	10.00	10.00	10.00	10.00	10.00	10
Rapeseed oil	4.15	4.70	5.50	6.00	6.30	6.02
Limestone (fine to coarse-40:60)	9.15	8.10	8.10	8.05	8.06	-
Rapeseed meal (CP 349)	-	-	-	-	-	5.00
Sunflower meal (CP 340)	-	-	-	-	-	5.00
Corn gluten	-	-	-	-	-	5.00
Potato protein	-	-	-	-	-	2.00
Monocalcium phosphate	1.28	1.28	1.29	1.30	1.31	1.52
Premix 0.5%	0.50	0.50	0.50	0.50	0.50	1.00
NaCl	0.18	0.19	0.20	0.20	0.19	0.61
NaHCO_3_	0.35	0.30	0.29	0.29	0.29	-
DL-Methionine (98%)	0.15	0.15	0.15	0.20	0.15	0.15
HCl-lysine (78%)	0.15	0.02	-	0.01	0.08	0.35
L-Threonine (98%)	0.05	0.02	0.02	0.01	0.04	0.02
L-Tryptophan (98%)	-	-	-	-	-	0.03
L-Valine (98%)	0.10	0.07	0.07	0.07	0.10	-
Metabolizable energy (MJ/kg)	11.61	11.62	11.68	11.64	11.62	11.80
Components%						
Crude protein	16.36	16.42	16.43	16.49	16.41	17.02
Ca	3.5	3.53	3.52	3.5	3.5	4.3
P-available	0.39	0.39	0.39	0.39	0.39	0.46
Na	0.18	0.17	0.17	0.18	0.18	0.24
Cl	0.18	0.16	0.16	0.17	0.17	0.46
Lys. digest.	0.76	0.75	0.75	0.75	0.75	0.69
Met + Cys digest.	0.65	0.64	0.65	0.67	0.65	0.62
Thr digest.	0.53	0.53	0.54	0.54	0.54	0.49
Tyr digest.	0.16	0.17	0.17	0.17	0.17	0.15
Val Tot.	0.71	0.71	0.72	0.72	0.72	-
Arg Tot.	0.98	1.25	1.34	1.43	1.52	-
Linol. Acid	1.73	1.67	1.84	2.01	2.16	-

**Table 9:** Chemical composition of seeds of blue lupin (g/kg DM) ^1^.

Component	Narrow-Leaved Lupin	Soybean Meal
Dry matter	879	916
Crude protein	263	481
Starch	10.7	54.6
Neutral Detergent Fiber	211	237
Total oligosaccharides (RFO ^2^)	59.5	43.7
Alkaloids	0.41	ND ^4^
Total NSP ^3^	406	170
Soluble NSP	195	67

Each value represents the mean of two replicates. ^1^ DM—dry matter. ^2^ RFO—raffinose family oligosaccharides. ^3^ NSP—non-starch polysaccharides. ^4^ ND—not determined.

## Data Availability

The data will be available after the results publication in RepOD—the Open Data Repository.

## Supplementary Materials

The following supporting information can be downloaded at: https://www.mdpi.com/article/10.3390/molecules29163727/s1, Table S1: Analysis of variance results of dependence between the amino acids content in egg parts (A—yolk, B—albumen) and the combined effect of diet and day of sample collecting; Table S2: Comparison of the amino acids in the studied eggs to the recommended amino acids scoring for older child, adolescent and adults (FAO, 20130) [g/100 g of protein] A—in white, B—in yolk. Table S3: The calculation of the essential amino acids amount delivered to human organism by single egg.
