# Supplementary material for: Modification of the Protein Amino Acid Content in Hen Eggs as a Consequence of Different Concentrations of Lupine and Soy in Feed
Source: Molecules. 2024 Aug 6;29(16):3727. doi: 10.3390/molecules29163727 (PMC11357042; doi:10.3390/molecules29163727)
Supplement: Supplementary file 1 [file molecules-29-03727-s001.zip › molecules-3067313-supplementary.pdf]

Table S1 : Analysis of variance results of dependence between the amino acids content in egg parts (A – yolk, B – albumen) and the combined effect of diet and day of sample collecting.

A

Amino acids of yolk	Main effect: Diet x day (interaction)		
	Sum of squares	F	p
His	3.0190	8.523	0.000095
Ser	78.5178	218.9680	<0.0001
Arg	53.40018	80.4548	<0.0001
Gly	12.4216	3.367532	0.019109
Asp+Asn	85.1708	155.2155	<0.0001
Glu+Gln	207.3083	231.4885	<0.0001
Thr	21.42344	74.34296	<0.0001
Ala	33.13058	327.3557	<0.0001
Lys	6.28936	12.49995	0.000005
Tyr	2.58098	6.281004	0.000737
Ile	20.29534	42.99700	<0.0001
Leu	66.73197	230.4290	<0.0001
Pro	16.75596	230.6742	<0.0001
Phe	16.34911	59.30633	<0.0001
Cys	10.70207	85.3381	<0.0001
Met	3.633131	7.68596	0.000196
Val	19.19334	19.55409	<0.0001

B

Amino acids of albumen	Main effects: diet x day		
	Sum of squares	F	p
His	4.0528	43.73	<0.0001
Ser	13.387	18.07	<0.0001
Arg	14.960	11.216	<0.0001
Gly	7.4702	17.325	<0.0001
Asp+Asn	79.926	37.13	<0.0001
Glu+Gln	203.391	150.40	<0.0001
Thr	8.2323	44.42	<0.0001
Ala	9.722	90.74	<0.0001
Lys	8.4492	16.555	<0.0001
Tyr	7.591	22.35	<0.0001
Ile	21.0018	81.80	<0.0001
Leu	21.444	62.95	<0.0001
Pro	442.3051	9574.641	<0.0001
Phe	12.777	12.216	0.000006
Cys	110.2651	18575.72	<0.0001
Met	204.3900	12764.43	<0.0001
Val	7.591	22.35	<0.0001

Table S2. Comparison of the amino acids in the studied eggs to the recommended amino acids scoring for older child, adolescent and adults (FAO, 20130) [g/100g of protein] A – in white, B – in yolk

A

Diet	Amino acid								
	HIS	ILE	LEU	LYS	SAA*	AAA*	THR	TRP	VAL
1	3.25	4.06	6.75	4.16	4.21	10.26	4.22	1.40	5.10
2	3.34	4.24	7.02	4.18	5.02	11.15	4.23	1.36	5.74
3	1.87	3.45	6.20	3.84	4.93	9.24	3.75	1.36	4.18
4	3.70	5.82	9.59	6.00	7.45	12.48	5.38	1.22	8.02
5	3.37	4.99	7.97	5.11	4.66	11.53	5.19	1.20	6.29
C	2.68	4.06	6.86	4.44	4.82	9.76	3.82	1.38	4.96
**FAO/WHO**	**1.5**	**3.0**	**5.9**	**4.5**	**2.2**	**3.8**	**2.3**	**0.6**	**3.9**

B

Diet	Amino acid								
	HIS	ILE	LEU	LYS	SAA*	AAA*	THR	TRP	VAL
1	3.48	4.51	8.00	5.39	3.05	6.60	4.96	1.08	4.51
2	3.19	4.26	8.02	5.52	3.67	6.94	4.85	1.14	4.46
3	3.87	4.54	7.88	5.78	3.20	6.81	4.79	1.20	4.98
4	2.58	4.21	7.67	5.24	2.11	6.87	4.64	1.10	4.68
5	2.96	4.77	8.43	8.08	3.33	6.80	5.08	1.06	5.17
C	3.56	5.66	8.43	7.06	3.69	8.29	6.62	1.16	6.19
FAO/WHO	1.5	3.0	5.9	4.5	2.2	3.8	2.3	0.6	3.9

*SAA: Cys+Met

*AAA: Phe+Tyr+Trp

Table S3. The calculation of the essential amino acids amount delivered to human organism by single egg.

Diet	ΣEAA* in yolk [g/100 g of protein]	protein content in yolk [%]	protein content in single yolk [g/20 g]	ΣEAA [g] in one yolk	ΣEAA in albumen [g/100 g of protein]	protein content in albumen [%]	protein content in single albumen [g/30 g]	ΣEAA [g] in one albumen	ΣEAA in one egg [g/1 pcs]
D1	38.10	16.1	3.22	1.227	36.6	11.5	3.45	1.263	2.49
D2	38.10	16.4	3.28	1.250	39.14	11.4	3.42	1.339	2.59
D3	39.70	16.4	3.28	1.302	32.16	11.5	3.45	1.110	2.41
D4	35.70	16.4	3.28	1.171	49.21	11.6	3.48	1.713	2.88
D5	41.86	16.7	3.34	1.398	42.6	10.6	3.18	1.355	2.75
C	47.22	16.2	3.24	1.530	35.9	11.8	3.54	1.271	2.80

*ΣEAA – sum of essential amino acids
